# Management of medicines in an eye hospital

**Published:** 2023-05-22

**Authors:** Ushalini Rasiah, Dhivya Ramasamy

**Affiliations:** Manager: Quality, Aravind Eye Care System, Madurai, India.; Senior Faculty: LAICO, Aravind Eye Care System, Madurai, India.

**Medication management is essential for safe and effective eye care.**

Medication forms a large part of ophthalmic treatment – a significant proportion of patients who undergo outpatient consultation at tertiary eye care facilities and primary eye care facilities are prescribed medication.

So it is important that the management of eye medication is integrated into eye care services at all levels. Great advancements in ocular medication therapy have resulted in a wide range of medicines being available, each with a specific use. This complexity makes it crucial for hospitals to implement a systematic and safe approach to drug dispensing. Without proper protocols, errors in medication dispensing can have serious consequences for patients.^1^

## The hospital or clinic pharmacy

A hospital or clinic pharmacy is required to manage stock of medication for two distinct purposes:

1. Medicines used within the clinic for diagnosis and procedures. These include dilating drops, anaesthetic drops, and medication used for emergency treatment or during surgery.
2. Medicines prescribed and dispensed to the patient for use at home. Some hospitals will give the patient a prescription to take to a pharmacy in the community.

The medications stocked in a hospital-based pharmacy depends on the services provided. For example, primary and secondary care facilities will stock common ocular medication used for allergies and infections, and tertiary care facilities may stock a more complex variety of medications for conditions such as glaucoma, diseases of the uveal tract, etc.

It is important to have a holistic view of the medications that patients may need. For instance, although glaucoma is not treated at a primary eye care facility, it would be helpful to stock common glaucoma medicines so that patients are able to get refills locally.

## Standardisation

It is important to standardise the list of medications and the brands that a hospital will use or dispense. This allows better price negotiation and stock management, and can help to ensure the quality and efficacy of the drugs.

## Prescription writing

A legible and complete prescription is essential for safe drug dispensing.

Prescriptions should be written **legibly** – possibly in capital letters. Where possible, printed prescriptions are preferred.

Prescriptions must be **complete**. It must have the patient's name and medical record number, the name of the drug, the strength, dosage, and duration. Any special instructions should be clearly and unambiguously written. Prescriptions must also carry the doctor's name and signature, and the date.

Ensure that only authorised personnel are permitted to prescribe medication. If an assistant writes down a doctor's orders, a policy of reading back what was written will help to prevent errors.

Where possible, give patients a consolidated set of instructions that factors in drugs from previous prescriptions as well as those prescribed at the latest visit.

**Figure:**
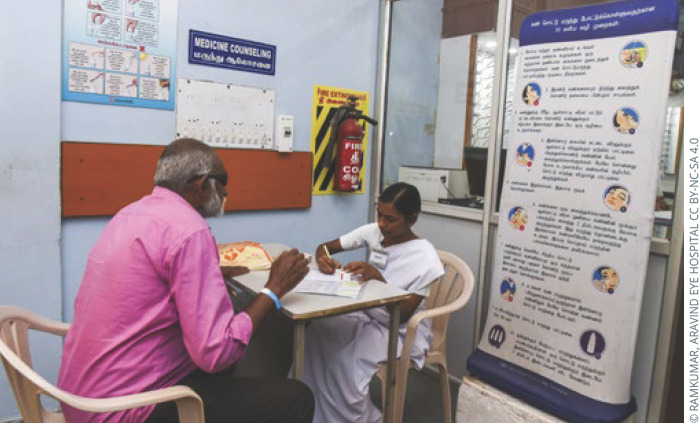
A pharmacy assistant counsels a patient about his eye medicines and how to use them. india

## Inventory management

Having an adequate supply of medicines in stock helps to ensure that patients receive treatment without delay. Medicines must be in date, and of good quality. It is therefore important to monitor stock levels (inventory) on a regular basis, and to check the quality of purchased medicines as they come in: check for any leakage, discoloured liquids, contamination, etc.

It is also important to develop good working relationships with a few reliable suppliers, as this can help hospitals to avoid fake drugs, negotiate better prices, and ensure sufficient stock levels. When there is a long-standing relationship, and mutual trust, suppliers may agree to replace medicines nearing their expiry date and respond to special drug requirements. Some suppliers may also provide stock on consignment, meaning that the hospital pays for medicines only when they have been sold.

Set up systems to ensure that stock is always dispensed according to a first-in, first-out method, so that medicines purchased first are dispensed first. Usually, this is done by stacking newly purchased packs at the back and dispensing packs from the front. A computerised inventory system can greatly enhance inventory management of drugs and alert staff about medicines nearing the expiry date.

**Figure:**
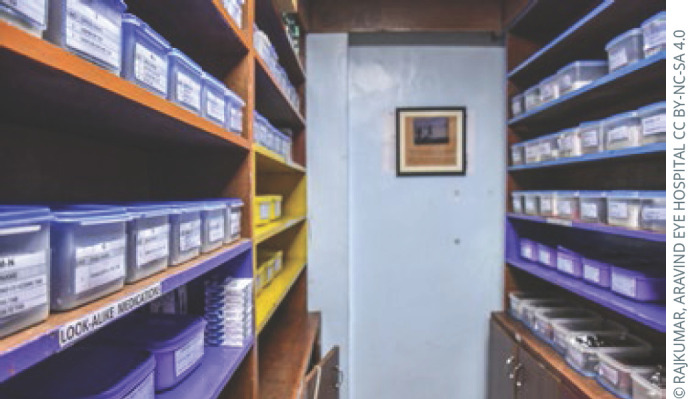
Look-alike medications are clearly labelled in this store room. india

## Storage and disposal

Always store medicines as recommended by the manufacturer. Make sure the storage area is well-lit, well-ventilated, and checked often to make sure no one is taking them without permission. Ensure that all medicines are kept at the correct temperatures.

Medicines that look alike and/or sound alike (also known as LASA) can result in dispensing and administering errors. Display a list of LASA drugs where pharmacists and other staff members can see it, and encourage the use of non-proprietary (generic) names for medicines. At Aravind, we train staff members to colour code LASA drugs and physically separate them within the storage area. Further guidance from the World Health Organisation is available at bit.ly/WHOlasa

Carry out regular audits to identify and remove expired drugs, and dispose of all medicines safely.

**Considerations when developing a standard drug list or formulary**

An essential first step is to set up standard treatment protocols and a standard operating protocol for patient examination.

Include medicines based on evidence of efficacy. The World Health Organization's Model List of Essential Medicines is a good place to start (sections 14.1 Diagnostic agents: Ophthalmic medicines and 21. Ophthalmological preparations). The latest version is available from https://bit.ly/WHO-em.

Ensure that the list not only has the “best” medication, but also includes affordable alternatives that are locally available. Review the list by continuously monitoring outcomes and looking out for new alternatives.

The standardised drug list should be communicated to the following people working in the hospital: doctors, counsellors (nurses or allied health personnel who counsel patients), pharmacists, and the people responsible for inventory management and purchasing. Some government-run eye facilities receive medications from the government without the option to choose brands or vendors. In such cases, hospital managers should advise purchasing officers about which drugs to order.

## Safe dispensing

Processes at the pharmacy must be standardised to ensure that staff always verify the prescription, ensure correct drug, strength and quantity, correct billing, and handing over with clear instructions. Medicine dispensing must be accompanied with detailed do's and don'ts – including how to open the eye drops bottle (not using a pin to perforate), hygiene, dosage and frequency to be followed, how long to wait between two medications, how and when to dispose of a medicine, and any special storage instructions (refrigeration, etc.).

## Enhancing adherence to medication

**Figure 1:**
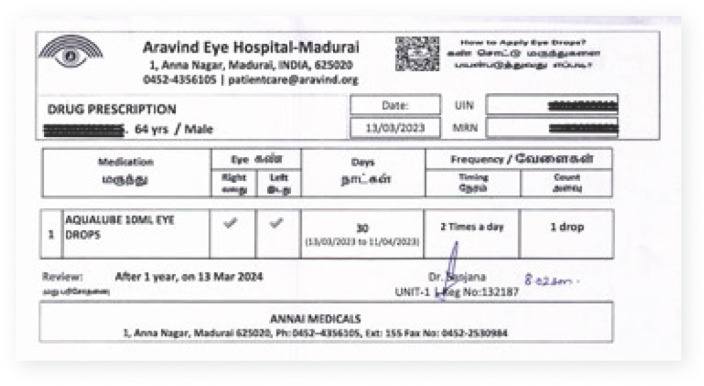
After getting their medicines, patients at Aravind Eye Hospital take the prescription home. The prescription shows the name of the eye drops, which eye(s) to use them for, how many days to use them, how often, and how many drops to use. The QR code (top right) links to a video that explains how to instil the drops.

Counselling during dispensing is important and should include information about why and how to take the medication. Where necessary, explain about drug-drug or food-drug interactions. The information should be provided in simple and clear language that the patient can understand. These oral instructions should be accompanied by printed or digital information for future reference. A QR code printed on the prescription can help patients and their family to access this information at a later date (see Figure 1).

**Figure 2:**
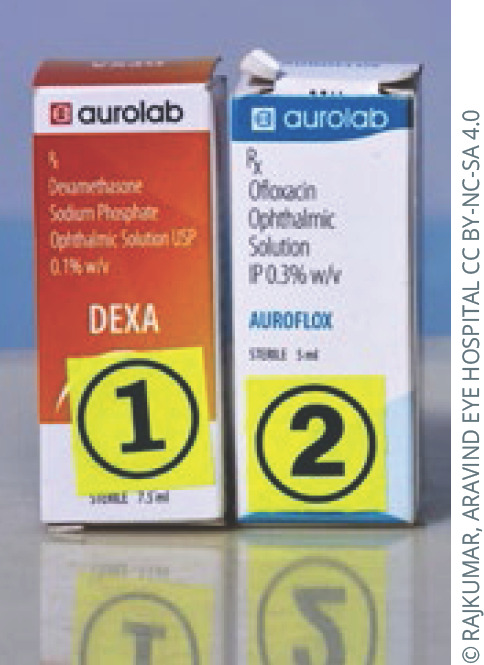
Medicines are clearly labelled with numbers, which are repeated in the instructions (or prescription) sent home with patients.

To make it easier for patients who have to use multiple medications, we add coloured or numbered labels (Figure 2). The prescription also refers to this number. This makes it easy for everyone to follow instructions, not just for those who are unable to read.

For patients taking medications for chronic conditions, counselling should include the importance of long-term adherence and instructions on how and where to get refills.

## Adverse drug reactions

Adverse drug reactions are undesirable events that follow the use of a drug. This can range from a simple rash to serious conditions, such as pulmonary oedema. Every drug reaction must be documented and analysed to understand whether the reaction is due to the medication itself, an interaction with another medicine, or whether there is an issue with a particular batch of medication.

Having a structured reporting system for this helps pharmacovigilance committees or local health care associations to track adverse drug reactions across the country, so they can make a collective decision on medicine about medicines and how to manage adverse reactions.
